# Dynamin- and Rab5-Dependent Endocytosis of a Ca^2+^-Activated K^+^ Channel, KCa2.3

**DOI:** 10.1371/journal.pone.0044150

**Published:** 2012-08-28

**Authors:** Yajuan Gao, Claudia A. Bertuccio, Corina M. Balut, Simon C. Watkins, Daniel C. Devor

**Affiliations:** Department of Cell Biology and Physiology, University of Pittsburgh, Pittsburgh, Pennsylvania, United States of America; Cinvestav-IPN, Mexico

## Abstract

Regulation of the number of ion channels at the plasma membrane is a critical component of the physiological response. We recently demonstrated that the Ca^2+^-activated K^+^ channel, KCa2.3 is rapidly endocytosed and enters a Rab35- and EPI64C-dependent recycling compartment. Herein, we addressed the early endocytic steps of KCa2.3 using a combination of fluorescence and biotinylation techniques. We demonstrate that KCa2.3 is localized to caveolin-rich domains of the plasma membrane using fluorescence co-localization, transmission electron microscopy and co-immunoprecipitation (co-IP). Further, in cells lacking caveolin-1, we observed an accumulation of KCa2.3 at the plasma membrane as well as a decreased rate of endocytosis, as assessed by biotinylation. We also demonstrate that KCa2.3 and dynamin II are co-localized following endocytosis as well as demonstrating they are associated by co-IP. Further, expression of K44A dynamin II resulted in a 2-fold increase in plasma membrane KCa2.3 as well as a 3-fold inhibition of endocytosis. Finally, we evaluated the role of Rab5 in the endocytosis of KCa2.3. We demonstrate that expression of a dominant active Rab5 (Q79L) results in the accumulation of newly endocytosed KCa2.3 on to the membrane of the Rab5-induced vacuoles. We confirmed this co-localization by co-IP; demonstrating that KCa2.3 and Rab5 are associated. As expected, if Rab5 is required for the endocytosis of KCa2.3, expression of a dominant negative Rab5 (S34N) resulted in an approximate 2-fold accumulation of KCa2.3 at the plasma membrane. This was confirmed by siRNA-mediated knockdown of Rab5. Expression of the dominant negative Rab5 also resulted in a decreased rate of KCa2.3 endocytosis. These results demonstrate that KCa2.3 is localized to a caveolin-rich domain within the plasma membrane and is endocytosed in a dynamin- and Rab5-dependent manner prior to entering the Rab35/EPI64C recycling compartment and returning to the plasma membrane.

## Introduction

The small conductance, Ca^2+^-activated K^+^ channel, KCa2.3 has been shown to play a critical role in a wide array of physiological processes [1]–[3]. The physiological response of any cell, with regard to ion channels, is determined by total current flow across the plasma membrane (I), which is in turn dictated by both the likelihood that the channels are in the open and conducting state, i.e., the open probability of the channel (P_o_) and the number of channels in the plasma membrane during stimulation (N) such that I α NP_o_. While a great deal has been learned about the regulation of KCa2.x and the related family member, KCa3.1 in terms of altering P_o_ [4]–[11] much less information exists regarding the mechanisms by which N is determined. We [9], [12]–[14] and others [15]–[17] have identified numerous motifs in the N- and C-termini of KCa family members which are required for the proper assembly and anterograde trafficking of these channels to the plasma membrane. Only recently, however, have reports begun to emerge that delve in to the mechanisms by which KCa2.x and KCa3.1 channels are endocytosed from the plasma membrane.

Recently, we published a series of studies demonstrating that KCa3.1 is rapidly endocytosed and targeted for lysosomal degradation [18]–[20] and that this lysosomal targeting requires ubiquitylation and USP8-mediated deubiquitylation [20]. Schwab and colleagues [21] have confirmed a rapid endocytosis of KCa3.1 in migrating MDCK cells and suggested this was clathrin-dependent. In contrast, we demonstrated that KCa2.3 is rapidly endocytosed and recycled back to the plasma membrane in a Rab35/EPI64C/RME-1-dependent manner in both HEK cells and HMEC-1 endothelial cells [18]. Absi et al. [22] have further shown that KCa2.3 resides in a caveolin-rich membrane domain in endothelial cells, although the endocytosis from this domain was not assessed. Herein, we demonstrate that the endocytosis of KCa2.3 from the plasma membrane is dependent upon caveolin-1 and dynamin II as well as Rab5, and that perturbation of these pathways leads to increased plasma membrane KCa2.3 as a result of a reduced endocytic rate. Taken together with previous studies, we propose that KCa2.3 is localized to caveolae, is endocytosed in a dynamin II- and Rab5-dependent manner after which KCa2.3 enters a recycling endosome compartment and is returned to the plasma membrane in a Rab35/EPI64C/RME-1-dependent manner [18].

## Materials and Methods

### Molecular Biology

The biotin ligase acceptor peptide (BLAP)-tagged KCa2.3 construct has been previously described [18]. For the generation of recombinant adenovirus (E1/E3-deleted) expressing BLAP-KCa2.3 the epitope-tagged channel was subcloned in to pAdlox. The myc-tagged KCa2.3 was a generous gift from J.P. Adelman (Vollum Institute, Oregon Health Sciences University), whereas the WT and K44A GFP-tagged dynamin II constructs were generously provided by N.A. Bradbury (Chicago Medical School). Rab5 was obtained as Addgene plasmid 14437 as provided by A. Helenius [23] and DsRed-Rab5 was obtained as Addgene plasmid 13051 as provided by R.E. Pagano [24]. GTP-bound, dominant active (Q79L) and GDP-bound, dominant negative (S34N) Rab5 mutations were generated using the Stratagene QuikChange™ site-directed mutagenesis strategy (Stratagene, La Jolla, CA). The fidelity of all constructs utilized in this study were confirmed by sequencing (ABI PRISM 377 automated sequencer, University of Pittsburgh).

### Cell Culture

Human embryonic kidney (HEK293) cells were obtained from the American Type Culture Collection (Manassas, VA) and cultured in Dulbecco’s modified Eagle’s medium (DMEM; Invitrogen, Carlsbad, CA) supplemented with 10% fetal bovine serum and 1% penicillin-streptomycin in a humidified 5% CO_2_/95% O_2_ incubator at 37°C. Cells were transfected using LipofectAMINE 2000 (Invitrogen) following the manufacturer’s instructions.

Wild-type (WT) and caveolin-1-deficient [Cav-1(−/−)] mouse embryonic fibroblasts (MEFs) cells were kindly provided by Dr. Carolyn Coyne (University of Pittsburgh) and cultured in high glucose Dulbecco’s modified Eagle’s medium supplemented with 10% fetal bovine serum and 1% penicillin-streptomycin in a humidified 5% CO_2_/95% O_2_ incubator at 37°C. WT and Cav-1(−/−) MEFs cells were infected with a recombinant adenovirus expressing BLAP-tagged KCa2.3 (BLAP-KCa2.3-Ad) generated by the University of Pittsburgh Vector Core. Brieftly, MEFs cells were washed three times with 37°C PBS, followed by incubation with BLAP-KCa2.3-Ad for 30 min at 37°C. Then, cells were washed once with PBS and allowed to recover until the next day in normal growth media.

### Antibodies

α-KCa2.3 Ab was obtained from Chemicon (Temecula, CA), α-myc Ab (9E10) was obtained from Covance (Richmond, CA), α-Rab5 Ab was obtained from Abcam (Cambridge, MA), α-GFP Ab was obtained from Santa Cruz Biotechnology (Santa Cruz, CA), α-tubulin Ab was obtained from Sigma-Aldrich (St. Louis, MO) and α-V5 Ab was obtained from Invitrogen.

### Biotinylation of KCa2.3 Using BirA

Biotin ligase (BirA) was expressed from pET21a-BirA (generously provided by Dr. Alice Y. Ting, Massachusetts Institute of Technology, Cambridge, MA) in *E. coli* as previously described [18]. HEK293 or WT and Cav-1(−/−) MEFs cells expressing BLAP-tagged KCa2.3 were incubated in PBS containing BirA (0.03 µg/µl), 10 mM ATP, 50 µM biotin, 1 mM Ca^2+^ and 2 mM Mg^2+^ for 30 min at room temperature. The cells were then washed 3x with PBS to remove the BirA and incubated in PBS containing 1% BSA plus appropriately fluorescently tagged streptavidin-Alexa488 or streptavidin-Alexa555 (0.01 mg/ml) for 10 min at 4°C. The cells were extensively washed with PBS/BSA to remove unbound streptavidin and then either incubated for various periods of time as indicated in the text at 37°C or immediately fixed and permeabilized with 2% paraformaldehyde plus 0.1% Triton X-100. BLAP-tagged KCa2.3 was also co-expressed with DsRed-tagged WT, S34N or Q79L Rab5. In other experiments, BLAP-KCa2.3 and GM1 (a marker for lipid rafts) were simultaneously labeled at the plasma membrane using streptavidin-555 and the cholera toxin B-subunit (CTX-B) conjugated to Alexa488 (1ug/ml) for 30 min at 4°C, respectively [25], [26]. Nuclei were labeled with DAPI. Cells were imaged by laser confocal microscopy using an Olympus FluoView 1000. To ensure maximal X-Y spatial resolution, sections were scanned at 1,024×1,024 pixels, using sequential three-color image collection to minimize cross talk between the channels imaged.

### Live-cell Confocal Microscopy

HEK293 cells were grown in a 35 mm glass-bottom dish (Mat-Tek Co) and co-transfected with BLAP-KCa2.3 and GFP-tagged WT dynamin II. Cells were incubated 10 min at 4°C with streptavidin-Alexa555 to label cell surface KCa2.3, as above and then returned to normal media. The dishes were then immediately transferred to a heated (37°C) chamber and imaged by laser confocal microscopy using an Olympus FluoView 1000 with 60x oil objective (plan apochromat, numerical aperture 1.4). 30–60 images were taken at 50 sec intervals. Images were processed using FV10-ASW version 02.01 (Olympus) and Photoshop version CS4 (Adobe).

### Total KCa2.3 Plasma Membrane Expression and Endocytosis Assay

Cell surface proteins, including KCa2.3, were biotinylated using EZ-Link Sulfo-NHS-SS-Biotin (Thermo Scientific, Rockford, IL) for 30 min at 4°C, after which the un-reacted biotin was quenched (PBS plus 1% BSA). For analysis of plasma membrane expression these samples were then immediately subject to cell lysis (50 mM HEPES pH 7.4, 150 mM NaCl, 1% v/v Triton X-100, 1 mM EDTA containing Complete™ EDTA-free protease inhibitor cocktail mix, Roche Applied Science, Indianapolis, IN) and streptavidin-agarose (Sigma-Aldrich) pulldown following normalization of protein concentrations and finally SDS-PAGE analysis. To evaluate endocytosis, the biotinylated samples were warmed to 37°C to allow channel endocytosis for various periods of time, as indicated. The remaining cell surface biotin was stripped using MESNA (100 mM sodium 2-mercaptoethanesulfonate, 50 mM Tris, 100 mM NaCl, 1 mM EDTA, 0.2% BSA) after which the cells were lysed and the protected (endocytosed) biotin-tagged channels subject to pulldown using streptavidin-agarose following normalization of protein concentrations. The endocytosed samples were compared to samples which were either biotinylated and immediately subject to lysis and streptavidin pulldown (total cell surface KCa2.3) or biotinylated and immediately stripped to confirm the efficiency of the MESNA stripping step (strip control). Proteins were resolved by SDS-PAGE (8% gel) and transferred to nitrocellulose for immunoblot analysis.

**Figure 1:**
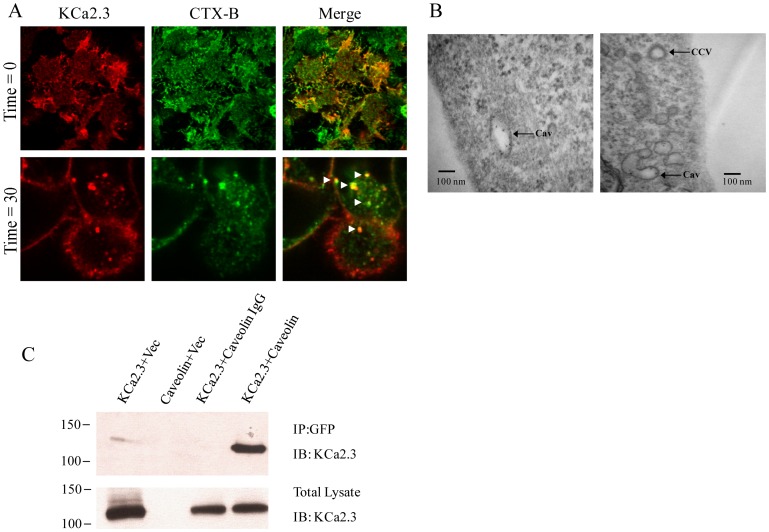
KCa2.3 is located in a caveolin-rich plasma membrane domain. **A.** BLAP-tagged KCa2.3 was expressed in HEK293 cells and labeled at the plasma membrane with streptavidin-Alexa555 (left panel), while lipid rafts were localized using the cholera toxin B-subunit (CTX-B) labeled with Alexa488 (middle panel). **Top Panels.** Single confocal section at the interface where the cells are attached to the glass coverslip showing extensive co-localization between KCa2.3 and CTX-B (merge) at T = 0 min. **Bottom Panels.** Single mid-plane confocal section after 30 min at 37°C showing endocytosed KCa2.3 co-localized with CTX-B (arrowheads in merge), indicating KCa2.3 is endocytosed in caveolae with CTX-B. **B.** Transmission Electron Micrograph (TEM) of Quantum Dot-labeled KCa2.3 following endocytosis for 30 min at 37°C. Electron-dense Quantum Dots are observed in structures consistent with caveolae (Cav). A clathrin coated vesicle (CCV) is indicated for comparison. **C.** Co-IP of KCa2.3 with GFP-tagged caveolin-1 was carried out in HEK293 cells as described in the Methods. Caveolin-1 was immunoprecipitated using an anti-GFP Ab (lanes 1, 2, 4) or an anti-V5 Ab as IgG control (lane 3) and subsequently IB using an anti-KCa2.3 Ab. When only a single construct was transfected the empty vector (vec) was included to maintain the plasmid at the same final concentration. KCa2.3 was detected by IB in lane 4, confirming an association between KCa2.3 and caveolin-1 (Top Panel). Bottom Panel confirms expression of KCa2.3 in total lysate by IB (5 µg total protein loaded per lane). Data are representative of 3 experiments.

### Immunoprecipitations (IP) and Immunoblots (IB)

Our IP and IB protocols have been previously described [20]–[22]. Briefly, cells were lysed and equivalent amounts of total protein were precleared with protein G-agarose beads (Invitrogen), and incubated with the indicated antibody. A non-specific IgG was used as negative control. Immune complexes were precipitated with protein G-agarose beads, the proteins resolved by SDS-PAGE followed by IB. To eliminate interference by the heavy and light chains of the immunoprecipitating antibody in the IP, mouse IgG Trueblot™ ULTRA (eBioscience, San Diego, CA) was used as a secondary antibody for the detection of immunoprecipitated proteins in the IB. Relative protein levels were quantified by densitometry using Quantity One software (Bio-Rad, Hercules, CA).

### Short-interfering RNA Treatment (siRNA)

The siRNA against the coding region of human Rab5 was obtained from Ambion (Grand Island, NY). The siGENOME Non-Targeting siRNA Pool no. 2 (Dharmacon Research, Chicago, IL) was used as control. HEK293 cells, stably expressing KCa2.3, were plated at ∼50% confluence and transfected with a mix of 50 nM Rab5 A, B and C siRNA duplexes using DharmaFECT 1, according to the manufacturer’s directions. Experiments were carried out 72 hrs post-transfection.

**Figure 2:**
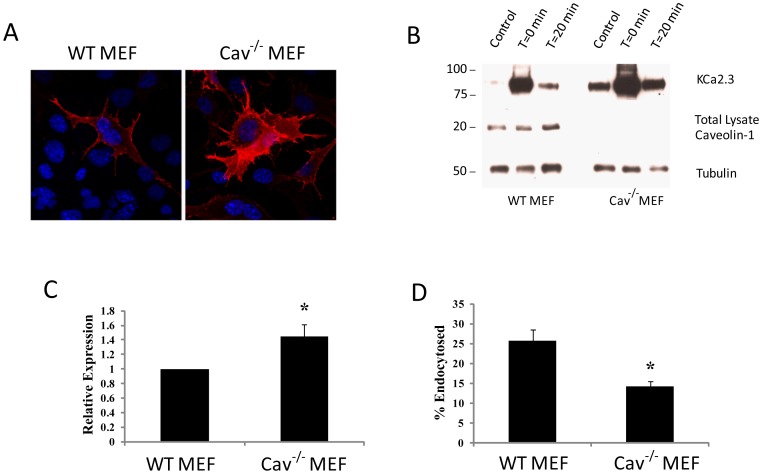
Absence of caveolin-1 decreases KCa2.3 endocytosis. **A.** Wild-type (WT) and caveolin-1-deficient [Cav-1(−/−)] mouse embryonic fibroblasts (MEFs) were infected with BLAP-KCa2.3-Ad, labeled at the plasma membrane with streptavidin-Alexa555 at 4°C after which the cells were imaged by confocal microscopy (see Methods). In Cav-1(−/−) MEFs, KCa2.3 cell surface expression appears to be significantly increased compared to WT MEF cells. **B.** Plasma membrane proteins were biotinylated using EZ-Link Sulfo-NHS-SS-Biotin (see Methods) after which the cells were incubated at 37°C for 20 min. Initial plasma membrane expression was determined by omitting the 37°C incubation step (T = 0 min, lane 2). The cell surface biotin which remained following endocytosis was stripped (MESNA) after which the endocytosed, biotinylated protein was pulled down using streptavidin-agarose, the proteins separated by SDS-PAGE and blotted for KCa2.3. The densitometries were determined and plotted in **C.** In the absence of caveolin-1, plasma membrane KCa2.3 was increased 1.44±0.16-fold (*n* = 3; p<0.05). Subsequently, the cell surface biotin which remained following endocytosis was stripped (MESNA) and KCa2.3 expression evaluated as above. The efficiency of stripping was determined by subjecting cells to MESNA in the absence of a 37°C endocytosis step (control, lane 1). Lane 3 demonstrates the amount of KCa2.3 endocytosed in 20 min (T = 20 min). The ratio between the amount of KCa2.3 detected following endocytosis (lane 3) to that at time 0 (lane 2) was determined by densitometry and plotted as % endocytosis in **D.** Knockout of caveolin-1 (Cav-1(−/−)) resulted in a significant decrease of KCa2.3 endocytosis, relative to WT MEFs (n = 3; *p<0.05). Caveolin-1 knockout was confirmed by IB. Densitometry is expressed as mean ± SEM. Tubulin was used as a loading control.

### Transmission Electron Microscopy (TEM) of Quantum Dot Labeled KCa2.3

HEK293 cells stably expressing KCa2.3 were grown on plastic coverslips for 24 hrs after which the channel was enzymatically biotinylated at the cell surface as described above, then labeled with streptavidin conjugated with QuantumDot-655 (Invitrogen). These QDots can be visualized in electron micrographs as dense geometric structures [19]. Following incubation at 37°C, the cells were fixed in 2.5% glutaraldehyde in PBS for 1 hr at room temperature. Samples were rinsed three times for five min in PBS and postfixed in 1% osmium tetroxide containing 1% potassium ferricyanide for 1 hour at room temprature and then rinsed three times in PBS. The cells were dehydrated in ascending grades of ethanol (30%, 50%, 70%, 90%) 10 min. each, and three changes of absolute alcohol for 15 min at room temperature. Samples were infiltrated three times with epon (1 hr each). Samples were embedded in epon overnight at 37°C and then 48 hrs at 65°C. Ultra thin sections were mounted on grids and stained in 2% uranyl acetate in methanol and then 1% lead citrate before imaging with a JEOL 1011 (Joel, Tokyo, Japan) electron microscope operated at an accelerating voltage of 80 kV.

### Chemicals

All chemicals were obtained from Sigma-Aldrich, unless otherwise stated.

### Statistics

All data are presented as means ± SEM, where *n* indicates the number of experiments. Statistical analysis was performed using a Student’s t test. To compare the normalized values of the IB band intensities, statistical analysis was performed using the non-parametric Kruskal-Wallis test. A value of p<0.05 is considered statistically significant and is reported.

**Figure 3:**
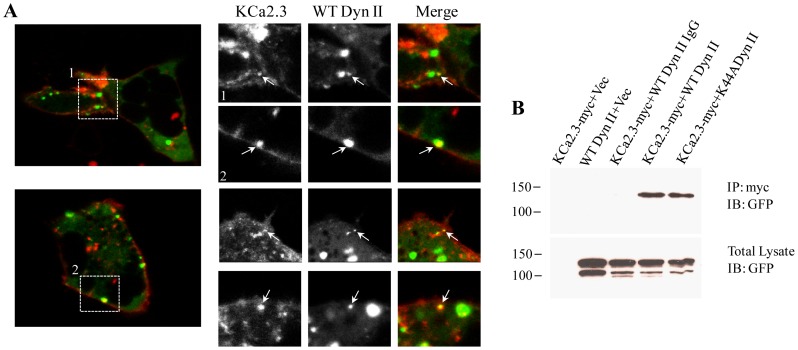
Endocytosis of KCa2.3 is dependent upon dynamin. **A.** GFP-tagged WT dynamin II and BLAP-tagged KCa2.3 were expressed in HEK293 cells. KCa2.3 was labeled at the plasma membrane with streptavidin-Alexa555 for 10 min at 4°C after which the cells were immediately imaged by live-cell confocal microscopy at 37°C (see Methods). **Left Panels** show individual images from two separate cells during the time course of the experiment. **Right Panels** show cropped images from these two cells (labeled 1 and 2) as well as cropped images from 2 additional cells. Arrows denote co-localization of KCa2.3 and WT dynamin II. **B.** Co-IP of myc-tagged KCa2.3 with GFP-tagged dynamin II. KCa2.3 was immunoprecipitated using an anti-myc Ab (lanes 1, 2, 4, 5) or an anti-V5 Ab as IgG control (lane 3) and subsequently IB using an anti-GFP Ab for dynamin II. WT and K44A dynamin II were detected by IB in lanes 4 and 5, respectively, confirming an association between KCa2.3 and dynamin (Top Panel). Bottom Panel confirms expression of GFP-tagged dynamin in total lysate by IB (5 µg total protein loaded per lane). Data are representative of 3 experiments.

## Results

### KCa2.3 is Localized to Caveolae in HEK293 Cells

Absi et al. [22] previously demonstrated that KCa2.3 is localized to a caveolin-rich, plasma membrane subdomain in endothelial cells and further demonstrated that KCa2.3 and caveolin could be co-IP. Thus, prior to evaluating downstream endocytic events we confirmed that KCa2.3 is localized to caveolae using the HEK293 model system. Initially, we addressed this by labeling BLAP-KCa2.3 at the plasma membrane using streptavidin-555 and used the cholera toxin B-subunit (CTX-B) labeled with Alexa488 as a marker for caveolae [25], [26]. As shown in Fig. 1A, KCa2.3 co-localized with CTX-B, confirming KCa2.3 is localized to caveolae in the plasma membrane. Further, after 30 min at 37°C, KCa2.3 and CTX-B were co-localized in endosomes (Fig. 1A), indicative of caveolae-dependent endocytosis of the channel. We also analyzed the endocytic compartment in to which KCa2.3 was initially retrieved by labeling KCa2.3 at the plasma membrane with streptavidin-Quantum Dots and allowing the channels to endocytose for 20 min at 37°C after which the cells were subject to TEM. As shown in Fig. 1B, KCa2.3 is observed in vesicles with a characteristic shape associated with endocytosed caveolae as opposed to the endosomes associated with clathrin-mediated endocytosis [27], [28]. Finally, we co-expressed KCa2.3 and GFP-tagged caveolin-1 and confirmed an association by co-IP as shown in Fig. 1C. Taken together, these results confirm that KCa2.3 is localized to a caveolin-rich subdomain of the plasma membrane.

To evaluate whether caveolin-1 is involved in the cell surface expression, as well as the endocytosis of KCa2.3, we utilized WT and Cav-1(−/−) MEF cells. We chose MEFs for these experiments as they offer a true null phenotype for caveolin-1, which was initially confirmed by immunoblot (Fig. 2B). Initially, we evaluated KCa2.3 expression at the plasma membrane in WT and Cav-1(−/−) MEFs by infecting with BLAP-KCa2.3-Ad and labeling plasma membrane channels using streptavidin-555. As shown in Fig. 2A, cell surface expression of KCa2.3 was apparently increased in Cav-1(−/−) MEFs compared to WT controls, as assessed by fluorescence labeling. This result was confirmed by biotinylation (T = 0 min.) as shown in Fig. 2B and C where KCa2.3 was increased an average of 1.44±0.16-fold (*n* = 3; p<0.05) in Cav-1(−/−) MEFs relative to WT MEFs; demonstrating an accumulation of KCa2.3 at the cell surface in the absence of caveolin-1. To evaluate whether expression of caveolin-1 is also required for the endocytosis of KCa2.3, we determined the fraction of channel endocytosed after 20 min in the presence or absence of caveolin-1 (see Methods). As shown in Figs. 2B and D, in WT MEFs 25.8±2.8% (*n* = 3) of KCa2.3 was endocytosed, whereas in the Cav-1(−/−) MEFs only 14.1±1.4% (*n* = 3; p<0.05) of KCa2.3 was endocytosed; confirming a role for caveolin-1 in the endocytosis of KCa2.3.

### KCa2.3 Endocytosis is Dynamin Dependent

Two of the major endocytic routes for proteins can be generalized as either clathrin-dependent or –independent and the clathrin-independent pathway can be viewed as either dynamin-dependent or –independent [29]. As the clathrin-independent and caveolar-dependent pathway has been shown to be dynamin dependent, we evaluated the role of dynamin II in the endocytosis of KCa2.3. Initially, we monitored the dynamics of KCa2.3 by live cell imaging to determine whether KCa2.3 was endocytosed in to dynamin II-containing vesicles. HEK293 cells were co-transfected with BLAP-KCa2.3 and GFP-tagged WT dynamin II and imaged as described in the Methods. In individual images taken throughout the time-course of the experiment, we observed that KCa2.3 and dynanin II co-localized in vesicles both near the plasma membrane as well as deeper in the cytoplasm (Fig. 3A), indicative of dynamin-dependent endocytosis. In addition, when we co-expressed myc- tagged KCa2.3 with either WT or K44A dynamin II, we were able to co-IP KCa2.3 with both WT and K44A dynamin II (Fig. 3B), suggesting that KCa2.3 and dynamin reside within the same compartment during the endocytic process.

**Figure 4:**
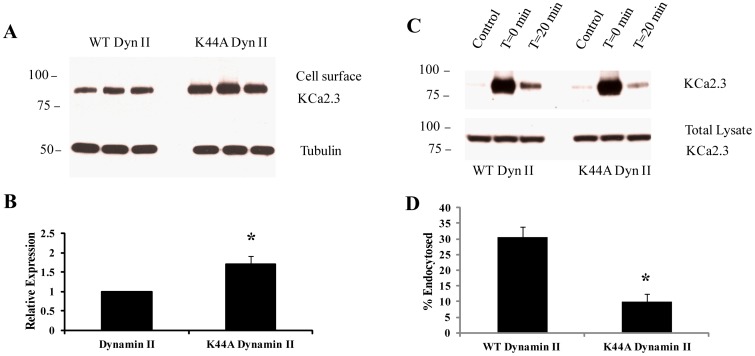
DN dynamin II (K44A) slows down endocytosis of KCa2.3. **A.** BLAP-KCa2.3 was co-expressed with either WT or K44A dynamin II and cell surface expression of the channel evaluated in triplicate as detailed in the Methods (top panel). Tubulin was used as a loading control (bottom panel). The data were quantified by densitometry for three separate experiments and plotted as shown in **B.** Expression of K44A dynamin II resulted in a significant increase in cell surface KCa2.3 expression (*p<0.05). **C.** BLAP-KCa2.3 was co-expressed with either WT or K44A dynamin II and endocytosis of the channel assessed. Plasma membrane proteins were biotinylated using EZ-Link Sulfo-NHS-SS-Biotin (see Methods) after which the cells were incubated at 37°C for 20 min. Initial plasma membrane expression was determined by omitting the 37°C incubation step (T = 0 min, lane 2). The cell surface biotin which remained following endocytosis was stripped (MESNA) after which the endocytosed, biotinylated protein was pulled down using streptavidin-agarose, the proteins separated by SDS-PAGE and blotted for KCa2.3. The efficiency of stripping was determined by subjecting cells to MESNA in the absence of a 37°C endocytosis step (control, lane 1). Lane 3 demonstrates the amount of KCa2.3 endocytosed in 20 min (T = 20 min). The ratio between the amount of KCa2.3 detected following endocytosis (lane 3) to that at time 0 (lane 2) was determined by densitometry and plotted as % endocytosis in **D.** Expression of K44A dynamin resulted in a significant decrease of KCa2.3 endocytosis, relative to WT dynamin (n = 3; *p<0.05). Densitometry is expressed as mean ± SEM.

As our results suggest a role for dynamin in the endocytosis of KCa2.3, we determined whether WT or K44A dynamin II would alter the plasma membrane expression of KCa2.3 using a standard biotinylation approach. As shown in Figs. 4A and B, expression of K44A dynamin II resulted in an approximately 2-fold increase in plasma membrane KCa2.3 expression compared to WT dynamin II (1.7±0.2-fold, p<0.05, *n* = 3), further suggesting that dynamin is required for KCa2.3 endocytosis. Finally, we directly evaluated the effect of dynamin II on KCa2.3 endocytosis by biotinylating KCa2.3 at the plasma membrane and determining the fraction of KCa2.3 endocytosed after 20 min in the presence of WT or K44A dynamin II (Figs. 4C and D). Similar to what we previously observed (18), KCa2.3 is rapidly endocytosed in the presence of WT dynamin II (30.5±3.4%). In contrast, K44A dynamin II dramatically slowed the endocytosis of KCa2.3 (10.1±2.3%; p<0.05, *n = *3), directly demonstrating that inhibition of dynamin function alters the endocytosis of the channel. Together, these results demonstrate a role for dynamin in the endocytosis of KCa2.3 in caveolae-containing vesicles.

### KCa2.3 Endocytosis is Rab5 Dependent

**Figure 5:**
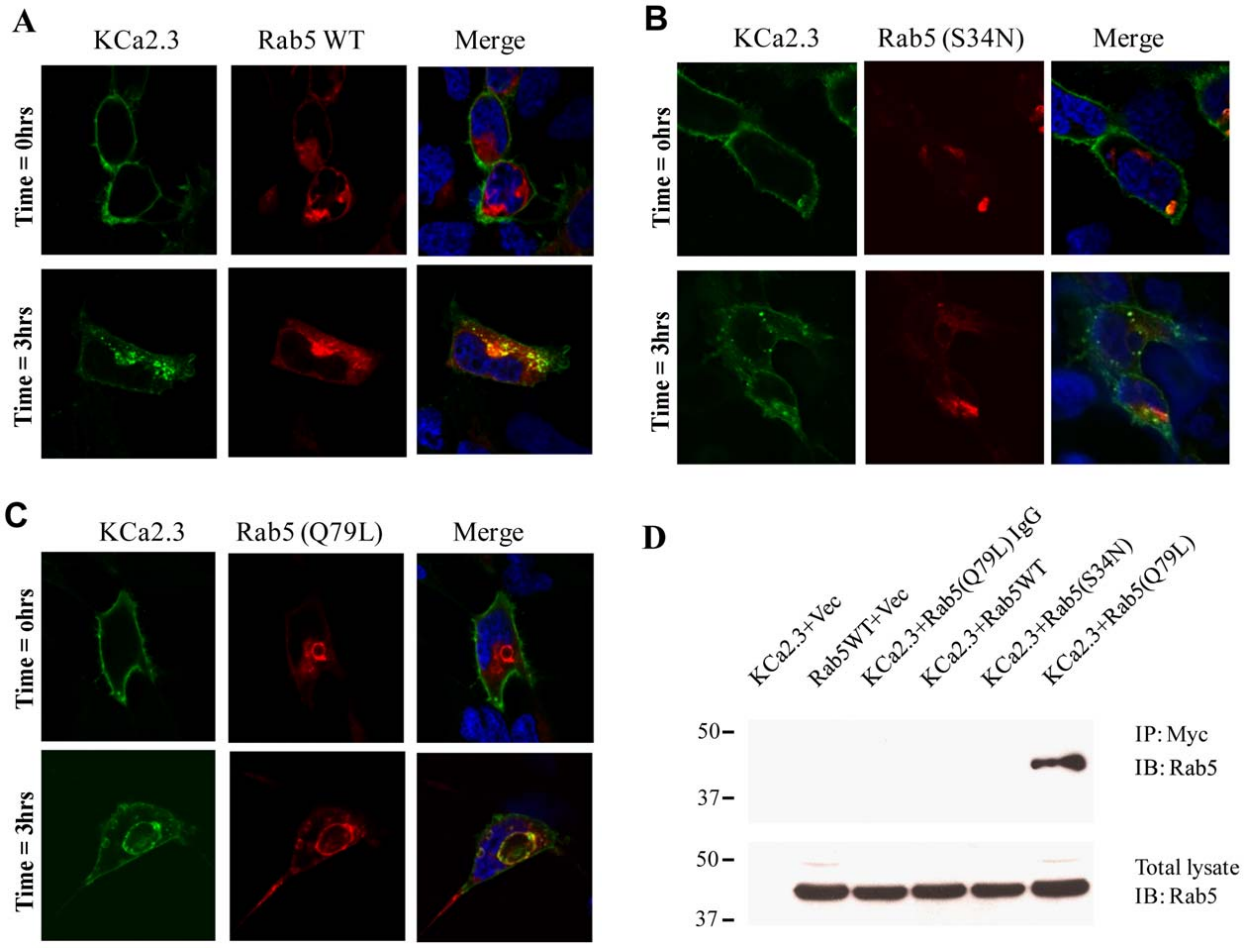
KCa2.3 enters Rab5 positive endosomes. BLAP-tagged KCa2.3 was co-expressed with DsRed-tagged WT (**A**), S34N (**B**) or Q79L (**C**) Rab5 in HEK293 cells. KCa2.3 was labeled with streptavidin-Alexa488 and localization evaluated by confocal fluorescence after 0 or 3 hrs at 37°C. KCa2.3 is localized to the plasma membrane at 0 hrs (upper panels) in each case, as expected. After 3 hrs (lower panels), endocytosed KCa2.3 co-localizes with WT Rab5 (A) as is clear in the merge (yellow color), whereas S34N appears to slow endocytosis (B). As is apparent, Q79L Rab5a induces the formation of intracellular vacuoles (**C**) and endocytosed KCa2.3 accumulates on these vacuoles; confirming co-localization of KCa2.3 and Rab5. **D.** Co-IP of myc-tagged KCa2.3 with DsRed-tagged Rab5. KCa2.3 was immunoprecipitated using an anti-myc Ab (lanes 1, 2, 4, 5, 6) or an anti-V5 Ab as IgG control (lane 3) and subsequently IB using an anti-Rab5 Ab. Q79L Rab5 was detected by IB (lane 6), confirming an association between KCa2.3 and Rab5. Note that an association was not detected between KCa2.3 and either WT or Q79L Rab5 suggesting these associations are very transient in nature. Bottom Panel confirms equivalent Rab5 expression (20 µg total protein loaded per lane). Data are representative of 3 experiments.

Both Rab5 and Arf6 have been shown to play important roles in the early endocytic steps of numerous proteins [30], [31] such that we determined whether either of these small GTPases are involved in the endocytosis of KCa2.3. Initial expression studies using Arf6 resulted in no clear alterations in KCa2.3 plasma membrane expression or endocytosis/localization and thus this small GTPase was not further evaluated (data not shown). The role of Rab5 on the endocytosis of KCa2.3 was initially evaluated by co-transfecting either WT DsRed-Rab5 or the S34N dominant negative or Q79L dominant active mutants with BLAP-KCa2.3 and determining the localization of the channel following labeling at the cell surface and then allowing endocytosis to proceed for 3 hrs at 37°C. As shown in Fig. 5A, KCa2.3 co-localized with WT Rab5 after 3 hrs, whereas expression of DN Rab5 appears to slow endocytosis in these experiments (Fig. 5B). Importantly, expression of Q79L Rab5 resulted in KCa2.3 accumulating on the induced vacuoles following endocytosis (Fig. 5C), clearly demonstrating that KCa2.3 enters a Rab5 compartment during endocytosis. An association between KCa2.3 and Rab5 was confirmed by co-IP as shown in Fig. 5D. As shown, we were only able to co-IP KCa2.3 and Q79L Rab5 suggesting that the association between KCa2.3 and WT Rab5 is too transient to observe under these conditions, whereas the dominant active form of Rab5 allowed this association to become apparent.

**Figure 6:**
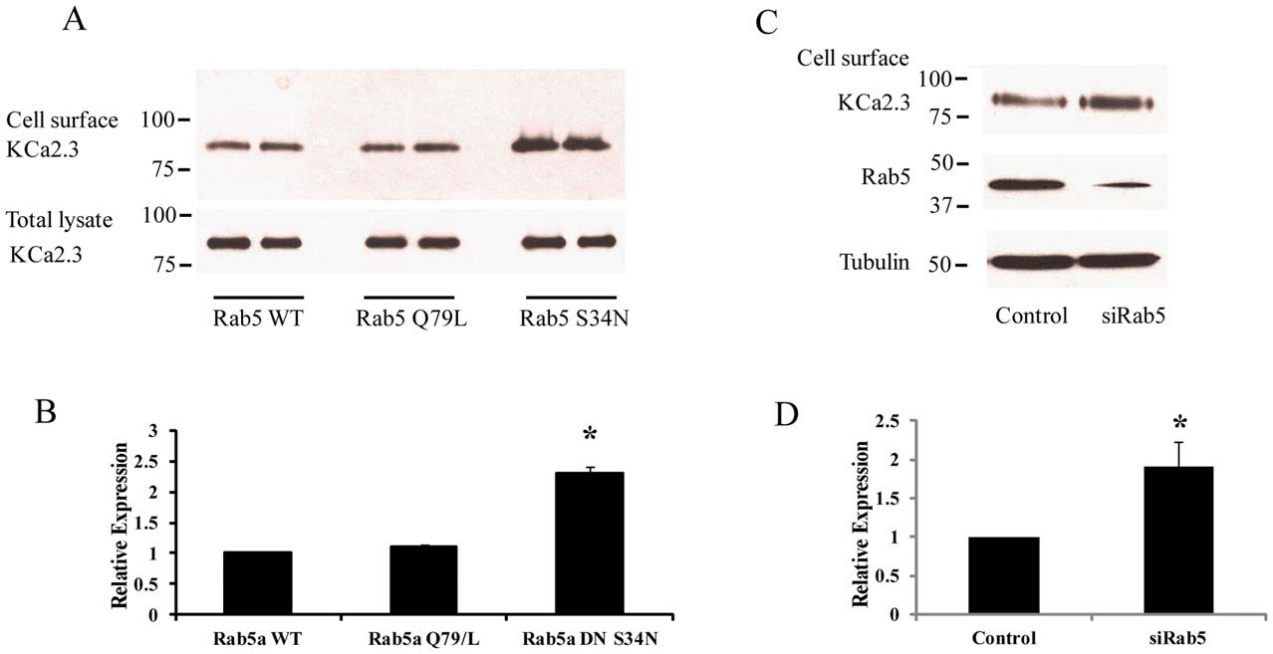
Cell surface expression of KCa2.3 is dependent on Rab5. **A.** BLAP-KCa2.3 was co-expressed with either WT, Q79L or S34N Rab5 and cell surface expression of the channel evaluated in duplicate as detailed in the Methods (top panel). Total KCa2.3 expression was also evaluated (total lysate) with no apparent effect of Rab5 expression (**A,** bottom panel). The data for cell surface expression was quantified by densitometry for three separate experiments and plotted as shown in **B.** Expression of S34N Rab5 resulted in a significant increase in cell surface KCa2.3 expression (*p<0.05). **C.** BLAP-KCa2.3 was co-expressed with either control or Rab5 siRNA (siRab5) and cell surface expression of the channel evaluated (top panel). Knockdown of Rab5 was confirmed by IB (middle panel). Tubulin was used as a loading control (bottom panel). The data for cell surface expression was quantified by densitometry for three separate experiments and plotted as shown in **D.** Knockdown of Rab5 resulted in a significant increase in cell surface KCa2.3 expression (*p<0.05). Densitometry is expressed as mean ± SEM.

If Rab5 is important for the endocytosis of KCa2.3 then it would be predicted that in the steady-state S34N Rab5 would increase KCa2.3 at the plasma membrane due to a decreased rate of endocytosis. This was confirmed by cell surface biotinylations, as shown in Fig. 6A. That is, expression of S34N Rab5 increased plasma membrane KCa2.3 expression 2.31±0.10-fold (p<0.05, n = 3) relative to WT Rab5 expression (Fig. 6B). In contrast, Q79L Rab5 had no effect on plasma membrane KCa2.3 expression (1.09±0.04-fold), indicating that the overall recycling pathway back to the cells surface has not been compromised in these studies. We confirmed these results by knocking down Rab5 expression using an siRNA-mediated approach. As shown, we achieved almost 70% knockdown of Rab5 relative to a scrambled siRNA control (Fig. 6C) which resulted in an approximately 2-fold increase in cell surface expression of KCa2.3 (1.90±0.32-fold, n = 3; p<0.05; Fig. 6D).

**Figure 7:**
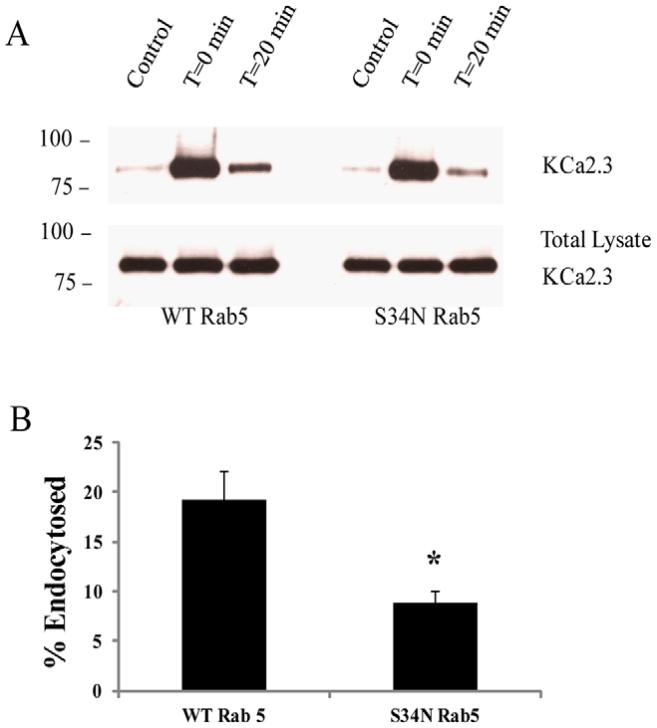
DN Rab5 (S34N) retards endocytosis of KCa2.3. **A.** BLAP-KCa2.3 was co-expressed with either WT or S34N Rab5 and endocytosis of the channel assessed. Plasma membrane proteins were biotinylated using EZ-Link Sulfo-NHS-SS-Biotin (see Methods) after which the cells were incubated at 37°C for 20 min. Initial plasma membrane expression was determined by omitting the 37°C incubation step (T = 20 min, lane 2). The cell surface biotin which remained following endocytosis was stripped (MESNA) after which the endocytosed, biotinylated protein was pulled down using streptavidin-agarose, the proteins separated by SDS-PAGE and blotted for KCa2.3. The efficiency of stripping was determined by subjecting cells to MESNA in the absence of a 37°C endocytosis step (control, lane 1). Lanes 3 demonstrates the amount of KCa2.3 endocytosed in 20 min (T = 20 min). The ratio between the amount of KCa2.3 detected following endocytosis (lane 3) to that at time 0 (lane 2) was determined by densitometry and plotted as % endocytosis in **B.** Expression of S34N Rab5 resulted in a significant decrease of KCa2.3 endocytosis, relative to WT Rab5 (*n* = 3; *P<0.05). Densitometry is expressed as mean ± SEM.

Our results suggest that S34N Rab5 slows down the endocytosis of KCa2.3 resulting in an increase in cell surface expression of the channel. Thus, we directly evaluated the effect of Rab5 expression on KCa2.3 endocytosis by biotinylating KCa2.3 at the plasma membrane and determining the fraction of KCa2.3 endocytosed after 20 min in the presence of WT or S34N Rab5. As shown in Fig. 7, in the presence of WT Rab5, 19.3±2.9% of KCa2.3 was endocytosed and entered the recycling pool after 20 min, similar to our results above as well as our previous observations [18]. In contrast, in the presence of S34N Rab5 only 8.9±1.2% (p<0.05, n = 3) of KCa2.3 was endocytosed after 20 min, directly demonstrating that S34N Rab5 decreases the endocytosis of KCa2.3. These results unequivocally demonstrate a role for Rab5 in the endocytosis of plasma membrane KCa2.3.

**Figure 8:**
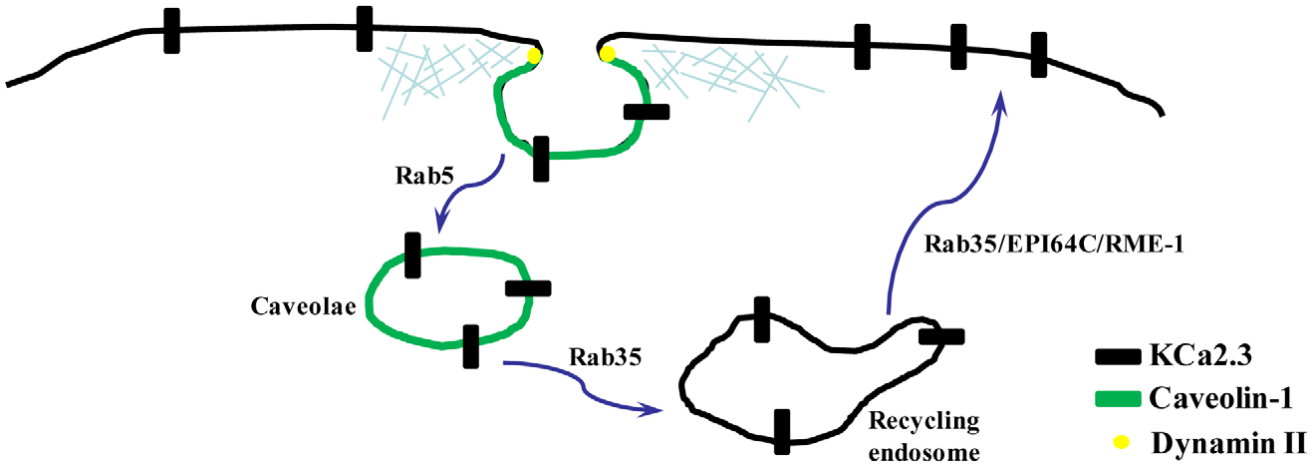
Schematic model for endocytosis and recycling of KCa2.3 based on data herein as well as our previous publication (18).

## Discussion

KCa2.3 plays a pivotal role in a host of physiological processes such that understanding not only the regulation and gating of this channel, but also its cell biology is imperative to fully appreciating its role in the cells and tissues where it is expressed. Clearly, both the open probability (P_o_) of a channel, as well as the number of channels (N) in the membrane, when an agonist is present, is deterministic in any physiological response. Very few studies, however, have explored the mechanisms by which KCa2.3 is either trafficked to the plasma membrane or the molecular mechanisms by which this channel is retrieved from the cell surface. Previously, we demonstrated that both an S4–S5 linker lysine [13], as well as charged amino acids in the S3 and S4 transmembrane domains [32], are required for the correct anterograde trafficking of KCa2.3 to the plasma membrane. Further, we demonstrated that misfolded KCa2.3 channels proteins are ubiquitylated and targeted to the proteasome in a derlin-1 and p97-dependent fashion [32]. In addition, Roncarati et al. [19] demonstrated an important role for the N-terminus, C-terminus and the calmodulin binding domain of KCa2.3 in the trafficking of KCa2.3 out of the endoplasmic reticulum. This latter observation is consistent with observations that the calmodulin binding domain is required for the correct trafficking of other members of the small conductance Ca^2+^-activated K^+^ channels [15], [16].

An important first clue to the mechanism by which KCa2.3 is retrieved from the plasma membrane came from the studies of Absi et al. [22], as they demonstrated that KCa2.3 is localized to caveolae in vascular endothelial cells. Recently, we demonstrated that KCa2.3 is rapidly retrieved from the plasma membrane and enters a recycling compartment from which the channel is returned to the plasma membrane with a time constant of ∼5 min [18]. We further showed that the recycling of KCa2.3 was dependent upon Rab35 as well as the Rab-GAP, EPI64C. Finally, we demonstrated that a small domain within the cytoplasmic N-terminus was critical to the recycling of KCa2.3 and that deletion of this domain altered the association of a component of the recycling endosome, RME-1 (EHD1) [18]. These studies left open the question of what are the initial endocytic steps involved in the trafficking of KCa2.3 from the plasma membrane to the recycling endosomes.

The endocytosis of plasma membrane, as well as the resident cargo, can be broadly divided in to phagocytosis or macropinocytosis, clathrin-mediated endocytosis or pathways which are generally referred to as clathrin-independent, including caveolae-dependent endocytosis. Initially, we demonstrate that KCa2.3 is localized to a caveolin-rich plasma membrane domain and that the channel is endocytosed in caveolae using fluorescence and TEM (Fig. 1A and B). Further, we demonstrate that KCa2.3 and caveolin co-IP (Fig. 1C). Finally, using caveolin-1-deficient MEFs cells, we observed that both KCa2.3 cell surface expression and endocytosis are dependent upon caveolin (Fig. 2). These results confirm and extend previous studies [22]; clearly demonstrating that KCa2.3 is localized to lipid rafts in a caveolin-rich domain of the plasma membrane and is endocytosed in to caveolae in a caveolin-1-dependent manner.

The endocytosis of cargo within lipid rafts can be either caveolin- and dynamin-dependent or dynamin-independent via non-caveolar intermediates [33]. Given the association of KCa2.3 with caveolin that we (Fig. 1) and others [22] have observed, we determined the role of dynamin in this process. As shown in Fig. 3A, KCa2.3 co-localizes with dynamin II in endocyted vesicles and co-IP confirms a close association between these proteins (Fig. 3B). Moreover, expression of a DN dynamin (K44A) resulted in an increase in steady-state plasma membrane KCa2.3 (Fig. 4A) as well as an inhibition of KCa2.3 endocytosis (Fig. 4C). These results further support the proposal that KCa2.3 is localized to lipid rafts and is endocytosed in a dynamin-dependent manner in caveolae.

A further division in the early endocytic fate of numerous proteins is based on the role that small GTPases play in the fusion of membranes and hence vesicle formation. The small GTPase, Arf6 has been shown to be involved in the early endocytosis and recycling of non-clathrin-dependent cargo [34], [35] while also being shown to play a role in Rab35-dependent recycling [36], [37]. As we previously demonstrated that KCa2.3 recycling is dependent upon Rab35 [18], we initially explored a role for Arf6 in the endocytosis of KCa2.3. However, DN Arf6 did not cause any apparent accumulation of KCa2.3 in an intracellular vacuolar compartment following endocytosis (data not shown) as would be expected [36], [37], suggesting that while the endocytosis/recycling of KCa2.3 is Rab35 dependent it is independent of Arf6. Rather, our studies support a role for Rab5 in the formation of KCa2.3 containing vesicles during endocytosis and subsequent delivery to the recycling endosomes. We demonstrate that KCa2.3 and Rab5 can be co-localized and also co-IP (Fig. 5A and D), indicative of these proteins being within the same vesicular compartment. In this regard, during steady-state endocytosis we observe approximately 20–30% of KCa2.3 in an intracellular compartment (Fig. 7A), which would include both early endosomes and recycling endosomes as well as any off-pathways, which have not been characterized to date. However, we were only able to co-IP KCa2.3 with dominant active Rab5, which results in enlarged endosomes such that KCa2.3 is trapped in an intracellular compartment associated with Rab5 [38]–[40]. It is well-known that Rabs associate with membranes in a GTP-dependent manner, whereas the hydrolysis of GTP to GDP results in the Rab dissociating from the membrane in to the cytosol [41]. Thus, our inability to detect an association between KCa2.3 and the DN Rab5 (S34N; GDP-bound) is expected due to its cytosolic localization. We speculate that our inability to detect an association between WT Rab5 and KCa2.3 is due to the transient nature of this association or the fraction of KCa2.3 moving through this compartment is too small to be routinely observed. In contrast, the DA Rab5 (Q79L; GTP-bound) remains associated with the endosomal membrane for an extended period thus increasing the likelihood of observing this association. In addition to demonstrating an association between KCa2.3 and Rab5, we show that expression of a DN Rab5 or siRNA-mediated knockdown of Rab5 results in an increase in plasma membrane localized KCa2.3 (Fig. 6A and C) as expected if Rab5 plays a role in the endocytosis of this channel. Finally, we directly demonstrate that inhibition of Rab5 function resulted in a slowing of the endocytosis of KCa2.3 (Fig. 7A); unequivocally demonstrating a role for Rab5 in the endocytosis of KCa2.3.

The precise control of the number of any channel, including KCa2.3, at the plasma membrane, N, is a key component of any physiological response. Given the dynamic nature of KCa2.3 at the plasma membrane any change in the rate constants for endocytosis or recycling would be expected to have both rapid and significant effects on the number of channels in the membrane. Clearly, one potential modulator of these rate constants would be agonists working through changes in either intracellular Ca^2+^ or kinases. Indeed, activation of β1 adrenoceptors in amygdala pyramidal neurons triggers activation of the PKA pathway, which has been shown to interfere with KCa2.2 recycling, effectively sequestering the channels into the cytosolic compartment [42]. It is also interesting to speculate that pharmacological agents that activate Ca^2+^-activated K^+^ channels through either an increase in P_oMAX_ or change in Ca^2+^-affinity may also alter N by modulating channel activity. Thus, understanding the mechanisms underlying KCa2.3 endocytosis and recycling is critical to clarifying how these channels alter the physiology of an organ system. Based on recent reports, as well as the data herein, this is starting to come in to focus leading us to propose the following mechanism: KCa2.3 is localized to lipid rafts and is endocytosed in caveolae containing caveolin-1 in a dynamin-dependent manner. The small GTPase, Rab5 is required for membrane fusion associated with KCa2.3 endocytosis after which the channel-containing vesicles are fused with a Rab35-containing recycling compartment. Recycling back to the plasma membrane occurs rapidly, with a time constant of ∼5 min and is dependent upon Rab35 as well as the Rab-GAP, EPI64C. Finally, this recycling is dependent upon a small N-terminal domain whose absence leads to a decreased association with RME-1 (EHD1) and subsequent more rapid degradation [18] (Fig. 8). These results lead to several additional questions, including whether the endocytosis and recycling of KCa2.3 is associated with a cycle of ubiquitylation/deubiquitylation as has been described for other ion channels that enter the recycling pathway [43]–[45] and whether this post-translational modification is manipulated physiologically as a means of altering N. In addition, it will be important to define the components involved in removing KCa2.3 from the recycling pathway and to determine whether this channel is ultimately destined for ubiquitylation and lysosomal degradation in a manner analogous to what we recently described for KCa3.1 [20] or some other degradative fate.
